# Incidental Finding of an Asymptomatic Aortic Dissection in a Patient With Catheterization Failure

**DOI:** 10.7759/cureus.20984

**Published:** 2022-01-06

**Authors:** Asrar Ahmad, Wajeeha Aiman, Muhammad Ashar Ali, Abbas Shehade, Addi Suleiman

**Affiliations:** 1 Cardiology, Saint Michael’s Medical Center, Newark, USA; 2 Sleep Medicine, Beth Israel Deaconess Medical Center, Harvard Medical School, Boston, USA; 3 Internal Medicine, Beth Israel Deaconess Medical Center, Harvard Medical School, Boston, USA

**Keywords:** abdominal aortic aneurysm repair, heart catheterization, chest ct angiography, type a aortic dissection, aortic dissection diagnosis

## Abstract

Aortic dissection (AD) is an injury to the innermost layer of the aorta, leading to the formation of a false lumen. AD usually presents with tearing chest pain radiating to the back and is a medical emergency. Other common symptoms include abdominal pain, diaphoresis, loss of consciousness, shortness of breath, stroke-like symptoms, or leg pain. Here, we present a rare case of an incidental finding of asymptomatic AD on computed tomography angiography performed after cardiac catheterization failure. The patient had a history of aortic aneurysm, hypertension, and heart failure. Appropriate imaging should be performed to rule out the possibility of AD in patients with risk factors and cardiac catheterization failure.

## Introduction

Aortic dissection (AD) is an injury to the intima (the innermost layer) of the aortic wall, which allows the blood to flow in between the layers of the aorta and medial degeneration, leading to the formation of a false lumen [1]. AD is categorized into the following two main types by Stanford: type A involving the aortic root, ascending aorta, and arch of aorta, and type B involving the descending aorta. Stanford dissection type A is more common and has a high mortality rate compared to Stanford type B [2].

AD is a relatively rare disease with an estimated incidence of 3/100,000 in one year. It is more common in patients with old age, history of hypertension (HTN), smoking, cocaine use, syphilis, bicuspid aortic valve, aortic aneurysm, connective tissue diseases such as Marfan syndrome, Ehlers Danlos syndrome, and previous heart surgery [3]. Sudden onset and tearing chest or back pain is the most common presentation of acute AD. Other presenting features include abdominal pain, syncope, amnesia, paraplegia, stroke, or vomiting [4]. Completely asymptomatic AD is rare but can lead to life-threatening complications. This report reviews a rare case of asymptomatic type A AD in a patient with systolic heart failure and aortic aneurism, incidentally diagnosed on computed tomography angiography (CTA). In patients with risk factors of AD, clinicians should keep the possibility of asymptomatic AD in mind in patients so that appropriate testing with transthoracic echocardiography (TTE) or CTA can be conducted.

## Case presentation

A 61-year-old male presented to the cardiology department for his scheduled cardiac catheterization to evaluate the hemodynamics and functioning of the heart. He had a history of HTN, cardiomyopathy, ascending aortic aneurysm, and systolic heart failure with an ejection fraction of 20-25%. Two months ago, the aortic aneurysm was diagnosed on a CT scan without contrast. During catheterization, the physician was unable to access the left anterior descending (LAD) artery. No other symptoms were reported, including chest pain, nausea, vomiting, or shortness of breath. The patient denied any use of alcohol, tobacco, or illicit drugs.

**Figure 1:**
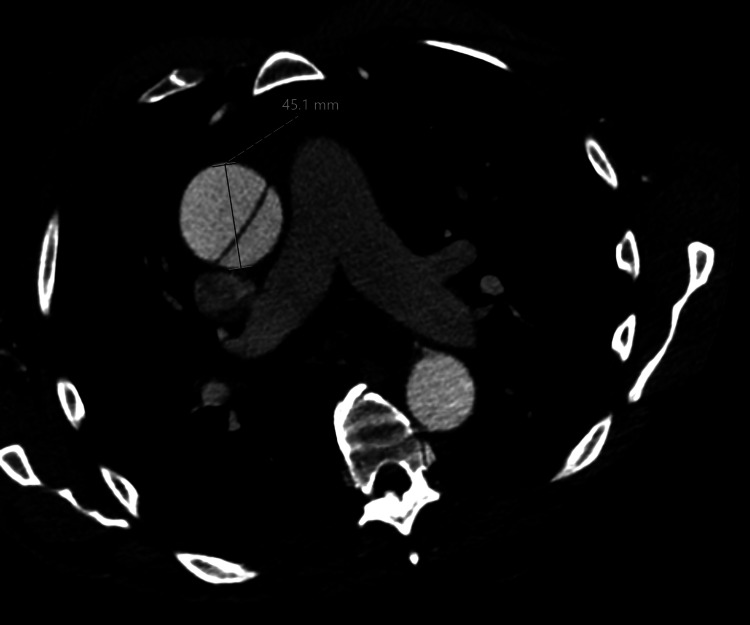
Aortic dissection at the aortic root on CTA. CTA: computed tomography angiography

The patient underwent CTA to determine the underlying cause of catheterization failure. CTA showed an intimal flap starting just superior to the right coronary and terminating within the aortic arch in the proximal portion of the right brachiocephalic artery (Stanford type A). Another small dissection flap was seen originating superior to the left coronary cusp extending approximately 1 cm superiorly. Marked aortic dilatation was also noted, which was more pronounced in the sino-tubular junction and mid ascending aorta measuring 5.3 cm and 4.5 cm, respectively. No occlusion or stenosis was detected in the evaluated coronary arteries. A calcified atherosclerotic plaque was noted in the mid LAD resulting in less than 50% stenosis. Small right and left-sided pleural effusions were also revealed (Figure 1).

The patient’s blood pressure was in the high 130s, for which he was given hydralazine. An electrocardiogram showed premature ventricular and atrial contractions; however, the patient remained asymptomatic. His troponin levels were normal.

Follow-up

Blood pressure was controlled to <120 mmHg on hydralazine and labetalol with monitoring and replacement of electrolytes. Bio-Bentall procedure for the aortic valve, aortic root, and ascending aorta was considered. However, the condition was carefully reviewed by the surgical team as the patient had multiple comorbidities. Moreover, he denied blood transfusions for religious reasons. An emergency surgical procedure was deferred because the condition was chronic. Elective surgical repair was done by the surgical team after satisfactory evaluation, and the patient developed no complications until the one-month follow-up.

## Discussion

**Figure 2:**
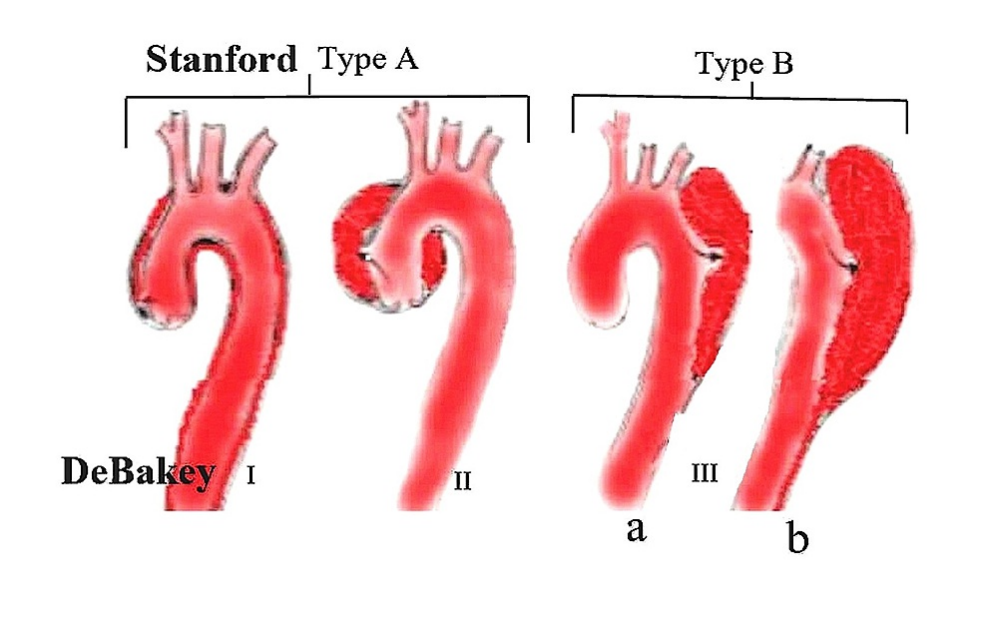
Types of AD. AD: aortic dissection

AD is divided into two main types by Stanford (type A and B) and three types by DeBackey (I, II, and III) (Figure 2).

AD is an emergency when presented acutely. It is associated with a high risk of morbidity and mortality, especially if left untreated [5]. Almost 80% to 90% of patients with AD die in two to three weeks if left untreated [6,7]. Although AD most commonly presents with sudden and tearing chest, abdomen, or back pain, 5-10% of the patients remain asymptomatic; moreover, AD is detected as an incidental finding [8]. Patients with painless AD can present with other symptoms such as syncope [9], vomiting, amnesia [10], paraplegia [11], and stroke. Dissection of the intima obstructs blood flow to multiple organs leading to the above-mentioned symptoms and tearing causes pain. Although rare, some patients remain entirely asymptomatic.

In our case, the patient presented to the office for a scheduled appointment and was diagnosed with an aortic aneurysm previously but was asymptomatic. The patient had systolic heart failure that can be exacerbated by asymptomatic AD. According to Januzzi et al., AD in heart failure patients is more likely to be asymptomatic and Stanford dissection type A [12]. HTN was a significant risk factor for AD in this patient, which was managed first. Approximately 70-80% of AD cases are associated with a history of HTN [13]. Our patient’s age and gender also placed him at a high risk of AD. Overall, 65% of all AD cases are reported in men, and 63 years is the mean age at diagnosis [14].

To our knowledge, more than 14 case reports of asymptomatic AD have been published, with Stanford type A as the most common type of AD [15-17]. However, in this case, the novelty was the catheterizing failure in this asymptomatic patient, and imaging was performed afterward. Therefore, asymptomatic AD should be kept in mind for catheterization failure in patients presenting with risk factors of AD.

With an increasing number of case reports, we propose careful TTE with a parasternal left ventricular view and suprasternal views or CTA in patients with an aortic aneurysm, which are crucial for diagnosing asymptomatic AD. Aortic aneurysm patients should be examined carefully to exclude any possibility of AD or aortic rupture, regardless of symptoms, as these are the two life-threatening complications of aortic aneurysms. Timely and radical intervention should be considered in asymptomatic AD patients as it can lead to aortic rupture, heart failure, or exacerbation of heart failure [12]. Marked aortic dilatation at the sino-tubular junction and mild dilatation at the ascending aorta is common and mostly silent until dissection or rupture.

The gold standard treatment of Stanford type A AD is surgery after controlling blood pressure with medication [18]. According to a study by Hagan et al., there was a decrease in mortality from 58% to 26% in surgically managed type A AD [2]. However, patients with advanced age, previous thoracic surgeries, and fatal comorbidities are at a high risk of perioperative mortality and should be managed carefully [19].

## Conclusions

We reported a rare case of asymptomatic AD. Although AD is a painful condition, chronic AD patients may remain asymptomatic and pain-free. Moreover, it can be seen in a patient with cardiac catheterization failure. Therefore, care should be taken to manage patients with risk factors of AD to exclude the possibility of AD. To avoid missing a diagnosis of asymptomatic AD, careful screening with TTE or CTA is required.
